# Pilot Study of a Web-based Decision Tool on Post-operative Use of Radioactive Iodine

**DOI:** 10.17925/EE.2017.13.01.26

**Published:** 2017-04-03

**Authors:** Shrujal S Baxi, Rachel Kurtzman, Anne Eaton, Eliza Dewey, Craig Bickford, Stephanie Fish, Leonard Wartofsky, R. Michael Tuttle

**Affiliations:** 1. Head and Neck Medical Oncology Service, Department of Medicine, Memorial Sloan Kettering Cancer Center, New York, New York, US; 2. Department of Medicine, Weill Medical College of Cornell University, New York, New York, US; 3. Department of Epidemiology and Biostatistics, Memorial Sloan Kettering Cancer Center, New York, New York, US; 4. The THANC Foundation, PO Box 1021, New York, New York, US; 5. Endocrinology Service, Department of Medicine, Memorial Sloan Kettering Cancer Center, New York, New York, US; 6. Division of Endocrinology, Department of Medicine, MedStar Washington Hospital Center, Washington, DC, US

**Keywords:** Thyroid cancer, radioactive iodine, thyroidectomy, decision-making, web-based

## Abstract

**Background**: The Thyroid Cancer Care Collaborative developed a web-based clinical decision-making module (CDMM) to inform risk-adjusted decisions on post-thyroidectomy radioactive iodine (RAI) use in papillary thyroid cancer (PTC). **Methods**: In a pilot study, we evaluated the CDMM in 19 PTC cases representing low- (five), intermediate- (seven) and high-risk (seven) disease. Two PTC experts and 10 PTC physicians reviewed cases and assigned risk level and RAI recommendation. The experts used a standard approach while the others used the CDMM. We assessed agreement between responses using a weighted Kappa. **Results**: Between experts, risk-assignment was concordant in 100%, 57% and 86% of low-, intermediate- and high-risk cases, respectively. Between CDMM users, risk-assignment was concordant in 100%, 29% and 14% in low-, intermediate- and high-risk cases, respectively (p=0.01). CDMM-assigned risk agreed with the expert-assigned risk in 100%, 25% and 0% of low-, intermediate- and high-risk cases, respectively (Kappa=0.69). For RAI use, the experts agreed in 15 cases while CDMM users agreed in eight. On further analysis, interpretation of extrathyroidal extension and lymph node staging led to discrepancies with the CDMM. **Conclusions**: For a web-based CDMM to accurately inform appropriate use of RAI in PTC, standard pathological and surgical reports are necessary.

The incidence of thyroid cancer is increasing at a rate of 7% a year; there were an estimated 62,450 cases diagnosed in the US in 2015 alone. The majority of this rise in incidence is explained by the growing number of incidentally detected well-differentiated, early-stage or ‘low-risk’ papillary thyroid cancers (PTCs).^3^ There is a growing awareness that many thyroid cancers may indeed be relatively benign in their behaviour and can be followed without any intervention.^4^ As a result, the management of differentiated thyroid cancer has undergone a major paradigm shift over the last two decades from a ‘one size fits all’ to a ‘risk-adapted’ approach. The American Thyroid Association (ATA) has led this effort by developing and publishing evidence-based guidelines on thyroid cancer management. The ATA guidelines incorporate tumour and patient characteristics to estimate the initial risk of recurrence (prognostication) and then use this information to inform recommendations on the use of adjuvant radioactive iodine (RAI) or remnant ablation therapy and the intensity and method of surveillance.^5^ One major goal of the ATA guidelines is to minimise potential harm from overtreatment for low-risk patients, while appropriately treating high-risk patients.

In well-differentiated thyroid cancer, adjuvant RAI is an effective method of attempting to address microscopic disease both in the thyroid bed (remnant thyroid) and distant metastatic sites. The phrases remnant ablation and adjuvant therapy are often used interchangeably, but there are distinct differences. A lower dose of RAI, 30 to 50 mCi (or 1,110 to 1,850 MBq) is used for remnant ablation while a higher dose, 100 to 150 mCi (3,700 to 5,550 MBq) is reserved for adjuvant therapy in patients deemed at high risk of micrometastatic disease.^6^ The use of RAI improves survival and decreases recurrence rates for high-risk patients with extensive disease, but does not change the already excellent prognosis of patients with low-risk disease.^7–9^ The use of post-thyroidectomy RAI has dramatically risen over the last three decades as part of the first course of therapy for thyroid cancer from 6.1% of cases treated in 1973 to 48.7% of cases treated in 2006.^10^ During the same time frame, an increasing proportion of patients have been diagnosed with low-risk thyroid cancer raising the question of benefit of added RAI therapy.^3^

Early recommendations on RAI use were shaped by retrospective studies completed in the 1970-80’s that reported decreased risk of recurrence in patients who received RAI therapy compared with those treated with surgery and thyroid suppression alone.^11,12^ In 2009, the ATA released their first guidelines with the definitive recommendation against RAI use in low-risk populations. A 2014 study showed that these 2009 guidelines only modestly reduced the use of RAI for the very-low-risk subgroup of patients.^13^ To improve dissemination of the new 2015 guidelines into clinical practice, the Thyroid Cancer Care Collaborative (TCCC) created 12 online clinical decision-making modules (CDMM), which ask clinicians to respond to clinical questions on a variety of clinical decisions. The CDMM used for this study (‘When to Administer Remnant Ablation’) uses responses to clinical questions to provide a guideline-based recommendation regarding administration of post-operative RAI. Similar to the *Adjuvant! Online* tool used by oncologists to determine post-surgical management in breast cancer, the CDMM incorporates demographic, surgical and pathological characteristics into algorithms to categorise risk of recurrence (low, intermediate and high) and to provide clinical guidance on recommendations regarding use of post-thyroidectomy RAI. In this pilot study, we aimed to assess the usability of CDMM in determining the use of RAI in a heterogeneous series of patients with resected well-differentiated PTC.

## Methods

The TCCC is an online Health Insurance Portability and Accountability Act (HIPAA)-compliant, cloud-based portal and registry that enhances the quality of care for patients with thyroid disease and improves the communication between physicians. The TCCC provides portability of information for patients and centralises all of their relevant clinical records. Data entry modules incorporate time-saving features and focus on specific aspects of the thyroid cancer management. The application is also an educational tool for both patients and physicians, delivering informative videos and CDMMs.

**Figure 1:**
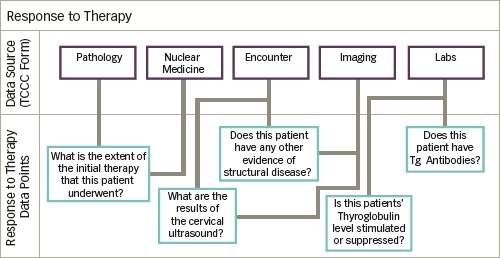
Flow of data incorporation and decision-making within the clinical decision-making module TCCC = Thyroid Cancer Care Collaborative

The TCCC developed the CDMMs in partnership with the experts who developed the most-recent version of the ATA guidelines. The overall process in the development of the CDMMs was first to isolate data pertinent to the specific clinical decision, and then to determine the necessary questions to ask to collect that data. Next, the applicable ATA clinical practice guidelines were reviewed to create a flow chart algorithm of decisions for the outcomes. Several iteration tests were run in order to verify the logic and to check for any gaps in the algorithm. Each outcome was cited and supported with a guideline footnote from the ATA clinical practice guidelines. The CDMM used for this study (‘When to Administer Remnant Ablation’) asks only five questions, one with a follow up if necessary, designed to isolate data points required to assess risk of recurrence based on the ATA guidelines ( see *Figure 1*). Then, based on these responses, the CDMM provides a risk level (low, intermediate or high) and one of three recommendations on use of RAI (yes, no or consider). If the answer is ‘consider’, there is some additional information on which groups may benefit from RAI, but no definitive conclusion due to a lack of consensus in the field.

The Institutional Review Board (IRB) at Memorial Sloan Kettering Cancer Center (MSKCC) approved this study. We identified study cases from an institutional database of 407 consecutive patients treated with a total thyroidectomy for PTC and stratified by ATA risk of recurrence from 2000 to 2002. We originally selected 20 cases from this dataset using a random number generator. Due to clerical inconsistency in one case upon later review, we only used 19 cases from this database (five from low-risk and seven each from intermediate- and high-risk) for analysis. Only cases with complete clinical information were included (e.g, surgical report, a pathology report, thyroid laboratories pre and post-surgery and records of all pre-surgical radiology).

For each selected case, a de-identified file containing the relevant clinical information that would have been available at time of a decision regarding adjuvant RAI was created. These files were provided to two experts (RMT, LW) who were asked to render a ‘yes’ or ‘no’ decision on the use of RAI for each case. The gold standard was deemed to be an agreement decision between two experts. Ten thyroid cancer fellows from endocrinology and surgery in fellowship at a specialised cancer centre were invited to participate (five endocrinology; five head and neck surgical fellows) and asked to use the CDMM to render a response of ‘yes’, ‘no’ or ‘consider’ regarding adjuvant RAI for the same 19 cases.

The number and percent of cases where all fellows agreed was described overall and by risk of recurrence based on ATA guidelines. The distribution of fellow’s responses was described for various types of cases. In cases of disagreement between the fellows, the question(s) on the CDMM causing disagreement was/were identified. Agreement between the fellows was assessed using weighted Kappa with equal weights, i.e. the distance from ‘no’ to ‘consider’ is equal to the distance from ‘consider’ to ‘yes’. (All statistical analysis was performed at R 3.1.1.R Foundation, Vienna, Austria) and the irr package was used.

## Results

### Case descriptions

The 19 cases randomly selected for this study represented a range of PTCs. There were five low-risk cases (26%), seven intermediate-risk cases (37%) and seven high-risk cases (37%). Of these cases, 10 patients were male (53%); seven patients were under 45 years of age (37%) at diagnosis.

### Provider decisions regarding use of adjuvant radioactive iodine therapy

**Figure 2:**
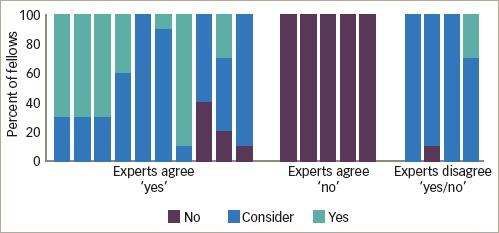
Figure 2: Agreement between experts and fellows on all cases

The experts had complete agreement in 15 out of 19 cases (79%) (see *Table 1*). There were 10 cases that were ‘yes’ agreement; five cases that were ‘no’ agreement. The experts agreed on 100%, 57% and 86% of low, intermediate- and high-risk cases, respectively (Kappa=0.68). There were four cases of ‘yes’/‘no’ disagreement between experts, three were intermediate-risk cases and one was a high-risk case. The 10 fellows agreed with each other in 42% (8/19) of cases. There were five cases of ‘no’ agreement, three cases of ‘consider’ agreement and zero cases of ‘yes’ agreement. The fellows agreed in 100%, 29% and 14% of the low-, intermediate-, and high-risk cases, respectively (p=0.01) (see *Figure 2*).

### Level of agreement by risk category

**Figure 3:**
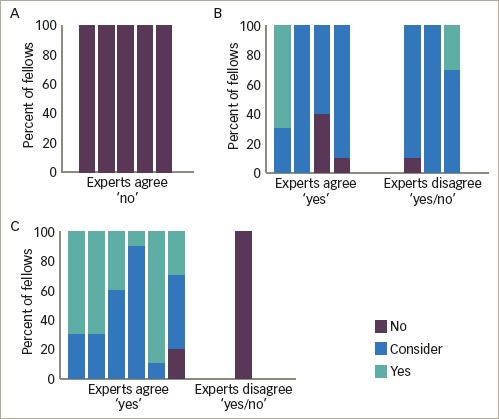
Agreement between experts and fellows, (A) low-risk cases, (B) intermediate-risk cases and (C) high-risk cases

In the 15 cases where a gold standard was established (agreement between the two experts), the fellows all agreed in 100% of the low-risk cases, 25% of the intermediate-risk cases and 0% of the high-risk cases (Kappa=0.69). The fellows agreed with the experts in 100% of the cases of ‘no’ agreement. In cases where the experts both concluded ‘yes’, the fellows concluded ‘yes’ an average of 38% of the time. The experts agreed in four intermediate-risk cases ‘yes’ give RAI, and the fellows agreed ‘consider’ in one of these cases. The experts agreed in six high-risk cases ‘yes’ give RAI, and the fellows did not agree on any of these cases (see *Figure 3*).

The experts agreed to give RAI in nine cases that the fellows disagreed on: Three intermediate-risk cases; six high-risk cases. Of the three intermediate-risk cases, the experts agreed ‘yes’ for, the fellows split between ‘consider’ and ‘no’ in two cases and ‘yes’ and ‘consider’ in one. Of the high-risk cases the experts agreed ‘yes’ for, the fellows split between ‘yes’ and consider’ in six cases, and between all three possible answers (yes’, ‘no’ and ‘consider’) in one.

There were four cases of ‘yes’/‘no’ disagreement between experts, three were intermediate-risk cases, and one was a high-risk case. In one of the intermediate-risk cases and the high-risk case, the 10 fellows agreed ‘consider’. In the other two cases the experts disagreed on, the fellows also disagreed, in one case splitting between ‘yes’ and ‘consider’, and in the other between ‘no’ and ‘consider’.

### Pathology reports

In a secondary analysis, when evaluating responses to feeder items in the CDMM, two main issues arose. First, there was disagreement about the presence of gross extrathyroidal extension. The CDMM, will always instruct ‘yes’ to give RAI if there is gross extrathyroidal extension. In all cases where there was ‘yes’/‘consider’ disagreement, the difference was attributable to the answer to gross extrathyroidal extension. The second cause of disagreement was response to N1a disease. In the absence of N1a disease, the CDMM will instruct ‘no’ to RAI. In the cases where there was ‘no’/‘consider’ disagreement, the only response that differed among fellows was the item on N1a disease.

**Table 1:** Agreement between experts and fellows

			Experts			
			Agree		Disagree	Total
			YES-RAI	NO-RAI	YES/NO	
**Disease specialists**	**Agree**	YES-RAI				
		NO-RAI		5		5
		CONSIDER	1		2	3
	**Disagree**	YES/NO				
		YES/CONSIDER	6		1	7
		NO/CONSIDER	2		1	3
		YES/NO/CONSIDER	1			1
**Total**			**10**	**5**	**4**	**19**

RAI = radioactive iodine

### Discussion

This pilot study confirmed that a web-based decision aid, the CDMM for RAI therapy, can be useful. However, the results highlight the challenges that clinicians caring for thyroid cancer face in deciding on post-surgical RAI therapy. Experts and fellows could all agree on ‘no’ for RAI for patients with low-risk disease, but there was definite disagreement, even among the experts themselves, for patients with intermediate- and high-risk disease. Upon evaluation of the fellows’ responses to the five individual items incorporated into the CDMM required to compute an ATA risk-level and make a recommendation for adjuvant RAI, two main issues explained almost all the disagreement: extrathyroidal extension and lymph node staging.

Identifying extrathyroidal extension can be extremely difficult, highlighting a potential roadblock in the utilisation of this tool. Non-fellows may not know that gross extrathyroidal extension into strap muscles is T3 disease, not T4a, unless specific structures are involved. As one of the experts pointed out, in some of the operative reports, surgeons had mentioned what would be considered gross extrathyroidal extension but there was no pathological confirmation. Specific circumstances from the randomly selected cases that represented ambiguous situations included: Extrathyroidal extension being identified on the non-dominant nodule; no mention of minor extrathyroidal extension; and staging of tumours with invasion of the tumour into a parathyroid gland. In recognition of the importance of standardised reporting of thyroid cancer, the Association of Directors of Anatomic and Surgical Pathology published guidelines in 2000 that could eliminate some of the variability seen in this study.^14^ Another area that presented clinical uncertainty was the distinction of N1a lymph nodes, which can be confusing if not explicitly stated by a surgeon or pathologist given the need to understand neck anatomy. Although we did not explicitly ask the experts and fellows to stage the disease, we suspect that there was some confusion interpretation between N1a and N1b disease.

It would appear that based on the 19 cases selected for this pilot study, the CDMM for post-treatment RAI may work best for cases that physicians would less need it for: Situations where administration of RAI is either never routine, or where it is always administered. For patients who fall into the ‘consider’ category, the likely cases that non-experts would go to a web-based tool for guidance, the CDMM did not clarify management. One suggestion would be to consider adding additional items for physicians to answer to reach a final decision that is ‘yes’ or ‘no.’ If the goal of the TCCC through the CDMM on post-operative RAI therapy and other important clinical decision tools is to help standardise care, then further items may be required to help clinicians reach a reproducible result or at least provide level of risk to facilitate conversations in unclear cases. Similar efforts have been practice changing in breast cancer, where studies have shown that Adjuvant! Online is consulted by an estimated 75% of oncologists in the US, and influences patient treatment.^15^

While this study evaluated a single CDMM, the TCCC has a larger goal: To improve communication between physicians by providing a single repository of clinical, patient and educational material that is tied to the individual patient and can be accessed by any and all providers. One goal of the TCCC database system is to standardise all narrative reports. Once physicians enter patient data into the TCCC’s discrete data points instead of open text boxes, reports are automatically generated. The TCCC includes narrative output reports on surgical management, pathology, ultrasound and imaging, initial presentation, follow-up exams and nuclear medicine. These standardised narrative outputs enable other members of the care team, specifically those of distinct fellows, to easily locate and understand the reported information, such as the extent of the extrathyroidal extension.

Feedback from this study has been taken back to the TCCC and a more-refined version of the CDMM on RAI therapy will be tested again using cases from after 2005 when our institutional pathology and operative reports for thyroid cancer were standardised to avoid confusion or misinterpretation of these key clinical features.

## Conclusions

The TCCC has developed a web-based clinical decision aid that is able to accurately direct clinicians to appropriate use of RAI therapy in some clinical scenarios. The ATA guidelines themselves leave a recommendation to ‘consider RAI ablation in intermediate risk’ scenario and do not provide a definitive recommendation that reinforces that decision aids are not meant to replace clinical experience and judgement but to help inform more evidence-based care. The goal for clinical guidelines and decision-making modules is to provide the clinician with the evidence available to guide a decision. With this goal in mind, another potential use of a tool like the TCCC CDMM for RAI ablation could also be to help educate trainees and future physicians on guideline-based care. This pilot study identified two problems limiting its broader use in its current format. First, a standardised pathological and surgical report would increase the reproducibility of outcomes between users and may need to be required for those that use the CDMM. Second, while highly effective in low-risk cases, the benefit of the decision aid is less clear on recommendations for intermediate- and high-risk cases and such a disclaimer might be helpful. Before implementation into clinical care, a much-larger validation study on the accuracy and effectiveness of such a tool are required.
